# Networks of neuroinjury semantic predications to identify biomarkers for mild traumatic brain injury

**DOI:** 10.1186/s13326-015-0022-4

**Published:** 2015-05-18

**Authors:** Michael J Cairelli, Marcelo Fiszman, Han Zhang, Thomas C Rindflesch

**Affiliations:** National Institutes of Health, National Library of Medicine, 38A 9N912A, 8600 Rockville Pike, Bethesda, MD 20892 USA; Department of Medical Informatics, China Medical University, Shenyang, Liaoning 110001 China

**Keywords:** Semantic predications, Semantic networks, Natural language processing, Degree centrality, Traumatic brain injury

## Abstract

**Objective:**

Mild traumatic brain injury (mTBI) has high prevalence in the military, among athletes, and in the general population worldwide (largely due to falls). Consequences can include a range of neuropsychological disorders. Unfortunately, such neural injury often goes undiagnosed due to the difficulty in identifying symptoms, so the discovery of an effective biomarker would greatly assist diagnosis; however, no single biomarker has been identified. We identify several body substances as potential components of a panel of biomarkers to support the diagnosis of mild traumatic brain injury.

**Methods:**

Our approach to diagnostic biomarker discovery combines ideas and techniques from systems medicine, natural language processing, and graph theory. We create a molecular interaction network that represents neural injury and is composed of relationships automatically extracted from the literature. We retrieve citations related to neurological injury and extract relationships (semantic predications) that contain potential biomarkers. After linking all relationships together to create a network representing neural injury, we filter the network by relationship frequency and concept connectivity to reduce the set to a manageable size of higher interest substances.

**Results:**

99,437 relevant citations yielded 26,441 unique relations. 18,085 of these contained a potential biomarker as subject or object with a total of 6246 unique concepts. After filtering by graph metrics, the set was reduced to 1021 relationships with 49 unique concepts, including 17 potential biomarkers.

**Conclusion:**

We created a network of relationships containing substances derived from 99,437 citations and filtered using graph metrics to provide a set of 17 potential biomarkers. We discuss the interaction of several of these (glutamate, glucose, and lactate) as the basis for more effective diagnosis than is currently possible. This method provides an opportunity to focus the effort of wet bench research on those substances with the highest potential as biomarkers for mTBI.

## Introduction

The diagnosis and treatment of traumatic brain injury (TBI) has received considerable attention. The military community may provide the biggest contribution to this interest because the signature injury of the wars in Iraq and Afghanistan is mild TBI (mTBI) [1]. mTBI is sometimes referred to as concussion, although the latter term is becoming less common in clinical and research contexts. The athletic community is also concerned with this condition, especially football and fighting sports, but also rugby, hockey, and soccer [2-8]. Although less newsworthy, falls cause the majority of head injuries in the US, with nearly 1.7 million TBI cases annually [9]. Worldwide, the annual incidence of mild TBI is estimated to be above 600 per 100,000 and, in addition to falls, motorcycle and bicycle accidents are also major causes [10]. As important as improvements in care are for veterans and athletes, such improvements can have a much broader impact on the health of communities around the world.

Although there is a need to improve the treatment of brain injury, perhaps the most significant hurdle is diagnosing mTBI. Current diagnostic standards are adequate for moderate and severe TBI because their signs and symptoms are more easily identifiable, but about 70-90% of TBI is mild, also known as concussion, and still difficult to recognize [10]. Additionally, the World Health Organization estimates that many mild injuries are not even seen by a health care practitioner because this lack of obvious and urgent symptoms fails to motivate patients to seek care [10]. Unfortunately, this does not mean that there are no long-term sequelae resulting from mTBI. According to current clinical research, mTBI sequelae include cognitive dysfunction, post-traumatic stress disorder, depression, anxiety, and dementia [2,11].

However, there are no currently accepted markers for clinical diagnosis of mTBI. Different organizations have created schematic tools for diagnosis, but these are subjective and the organizations do not completely agree on what constitutes a concussion [12]. For the greatest impact for military applications and throughout the world, as well as to minimize costs, a blood-based test would be ideal. Thus far such a test has not been found. There have been several candidate substances (S100B, neuron-specific enolase, glial acidic fibrillary protein, etc.), but none have succeeded for effective diagnosis of mild injury [13]. Because the search for a single biomarker has not succeeded, a composite panel may be an effective alternative. We present a method to help facilitate the identification of substances that have potential as biomarkers, which can then be validated experimentally.

As demonstrated with systems biology [14], the molecular interactions that occur after neurological injury are complex. There may be no serum value for any of the individual components of this complicated interplay that are specific to neural injury. However, some specific combination of these values included in a panel has much greater potential for diagnostic accuracy. The first step in investigating which substances belong in such a panel is to identify the potential candidates for inclusion. In this paper, we describe a methodology to provide a list of substances that is intended to establish a base of current knowledge and provide insight into the development of a biomarker panel for mTBI diagnosis. We apply natural language processing to MEDLINE citations to extract semantic predications, which we represent as a network of potentially relevant substances interacting with their physiological environment. These semantic predications are subject-relation-object triples, where the subjects and objects are UMLS concepts and the relation is derived from the UMLS Semantic Network as appropriate for a given concept pair. We then use network analysis techniques to identify a list that is focused on highly significant substances.

## Background

### Systems medicine

Our approach to diagnostic biomarker discovery was inspired by systems medicine, the application of systems biology to medicine. The underlying philosophy looks at biology as ‘information science’ and is concerned with the network of molecular interactions that define biological processes [14,15]. Additionally, disease states are viewed as a perturbation of these molecular networks [15]. In the case of traditional TBI biomarker discovery, the approach has been to seek an individual molecule to represent a disease state, while disregarding any notion of a network let alone its perturbation. Wang et al. describe this approach as pauciparameter, containing an inadequate amount of information and resulting in inadequate characterization [15]. The network must be considered as a whole, because a network perturbation does not necessitate that any of the individual molecules are outside of their normal serum measures, especially at early stages of disease, when prevention is still possible or treatment is optimal. They give prostate specific antigen for prostate cancer screening as an example of a failure of the traditional single marker, pauciparameter approach [15].

### Natural language processing

Natural language processing combines artificial intelligence and linguistic theory to extract meaning from text, using statistical machine-learning, hand-written rules, or a combined approach [16]. The data utilized in this study were provided by SemRep, which extracts semantic predications from all titles and abstracts in MEDLINE [17]. These predications take the form of a subject-predicate-object triplet. The subject and object are mapped to Unified Medical Language System (UMLS) concepts using MetaMap [18] and are stored with their UMLS semantic type, whereas the predicate is mapped to the UMLS Semantic Network [19]. This provides precise semantic meaning from the source text. For example, from the sentence in (1), SemRep extracts the predications in (2).

1. Basic science and clinical observations supportive of the role of endothelins in the spasm associated with stroke and subarachnoid hemorrhage are presented. (Pubmed ID 15281894)
2. *Endothelin ASSOCIATED_WITH Spasm*
  *Spasm ASSOCIATED_WITH Cerebrovascular accident*

The results of this process are stored in a predication database, SemMedDB [20], which has been used to support a range of biomedical information management research: identifying novel therapeutic approaches [21], labeling extracted information from clinical text [22], literature-based discovery [23-26], clinical information retrieval for physicians [27], retrieving clinical documents [28], abstraction summarization of biomedical texts [29], biological entity recognition [30], identifying disease candidate genes [31], support for cardiovascular clinical guidelines [32,33], interpreting microarray data [34], extracting research findings from literature [35], and supporting formal models of knowledge representation [36,22].

### Networks of semantic predications

**Figure 1:**
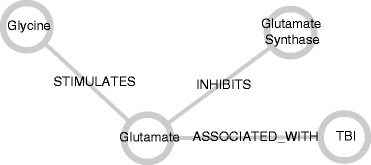
Network of glutamate predications. Subject and object concepts are represented as nodes and predicates are represented as edges. Glutamate is common to all three predications and is, therefore, the most highly connected node in the network.

Any concept in a set of predications can serve as either subject or object in various relationships. For example, one can imagine the concept *Glutamate* appearing in many predications similar to the following: *Glutamate ASSOCIATED_WITH Traumatic Brain Injury*, *Glutamate INHIBITS Glutamate Synthase*, or *Glycine STIMULATES Glutamate*. Similarly, any concept can have a set of relationships that include it as either the subject or object. Further, any set of predications can be represented as a network with each concept symbolized as a node and each relationship denoted by an edge (or arc) between the two nodes that represent its subject and object. A network containing the above predications is contained in Figure 1.

One of the goals of network theory is to establish significance of a given node or relationship. Degree centrality is based on the number of connections a node has and Zhang et al. [37] have shown that it is effective for identifying nodes in a graph that humans consider important. We have previously applied degree centrality to SemRep generated semantic predications to successfully summarize therapeutic studies [38]. For node (or vertex) *v*, the degree centrality is calculated by dividing the total number of nodes connected to *v*, *deg*(*v*), by the total number of nodes in the network other than *v*, *n*-1:

$$ {C}_d(v)=\frac{ \deg (v)}{n-1} $$

A simple means of judging the value of a given relationship is the frequency of the relationship, that is, a simple count of how many times it occurs in a given set. When using an automated tool, a single occurrence of a predication is much more susceptible to computational error than a predication with multiple instances. Therefore, a higher frequency may provide more confidence in the validity of the relationship, but at the same time, a high frequency is reflective of an abundance of assertions in the literature which is likely to be indicative of a well-known fact and may be less desirable for novel discovery.

### Incorporation of systems medicine, natural language processing, and network theory

This methodology combines ideas and techniques from systems medicine, natural language processing, and network theory. A network of relationships involving substances is created, but the data source is semantic predications from MEDLINE citations rather than genomic or other large-scale experimental data as have often been used for systems medicine. These semantic predications provide a computable form of the knowledge contained in MEDLINE that includes gene, protein, and metabolite relationships analogous to the experimental data traditionally used in systems medicine, as well as additional types of relations at the organism, system, organ, tissue, cell, and molecular level. Statistical approaches are often used to establish correlation and significance of different components in the experimental data of systems medicine, whereas a network of semantic predications provided by SemRep naturally expresses the network of interactions postulated by systems approaches. Network filtering techniques are used to further suggest significance of the individual concepts and their relationships. By coupling components from these three fields, a novel method of biomarker discovery is proposed.

### Related work

Several manual reviews have been undertaken to survey potential biomarkers for TBI [39,40] and more specifically mTBI [41-43]. These authors search for citations specifically detailing clinical research of mTBI biomarkers and therefore contain only potential biomarkers that have already been investigated. Another limitation of the studies is the small number of citations reviewed (ranging from 26 [42] to 107 [43]) due to the limitations of human review. Although no automated detection of potential TBI biomarkers exists in the literature, there are automatic systems to help diagnose other disorders, for example diabetes and obesity [44]. Although not related to mTBI, there is research related to the literature-based discovery of other types of interaction networks (though not specifically for biomarkers). One automatically generates an interaction network detailing gene involvement in vaccine-related fever using 170,000 citations from a PubMed search and a vaccine—specific ontology [45]. Another used citations containing the PubMed MeSH term *human* and containing sentences related to interferon-gamma, from which relationships were extracted and ranked using graph metrics [46]. Jordan et al. [47] present a keyword search method for identifying putative biomarkers for breast and lung cancer by searching for genes and proteins associated with a biological fluid keyword and either cancer. However, none of this work has made use of semantic predications, as we have, in the formation of an interaction network. There is a large body of work on literature-based discovery approaches many of which use SemRep semantic predications [26,48-54]. These approaches may generate systems for discovery [55-58] or are specific applications to predict various phenomena such as interactions between genes and proteins [46,59], cancer treatments [60,61], adverse drug reactions [49], drug-drug interactions [50], drug repurposing [51,62], asthma gene associations [63], treatments for neovascularization in diabetic retinopathy [52], relations between psychiatric and somatic diseases [64], genes related to reactive oxygen species and diabetes [65], and mechanisms for sleep disturbance [25] and the obesity paradox [53].

## Methods

**Figure 2:**
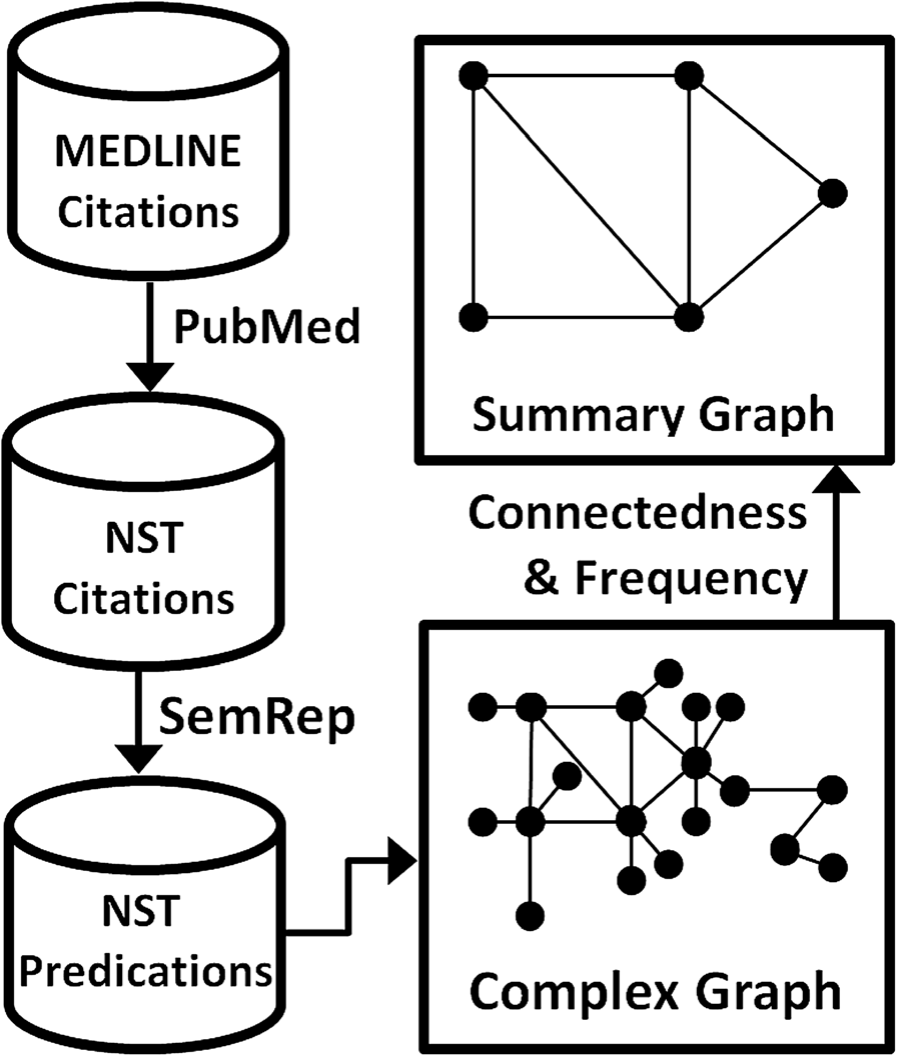
Overview of methodology. A PubMed search was used to find citations related to nervous system trauma (NST). SemRep extracted predications containing chemical substances from these citations, which were then arranged into a network. The network was filtered by connectedness (degree centrality) and frequency to provide a summary view of the most significant relationships.

As shown in Figure 2, citations related to nervous system trauma were retrieved from PubMed. From these, predications were extracted that contain a substance as the subject or object. These predications were organized into a single network which is then filtered to select for the most highly connected and frequent components. The substances included in this summary network serve as the list of potential mTBI biomarkers.

### Citation search

A PubMed search for all articles containing the MeSH term *Trauma, Nervous System* was used to generate a list of PubMed identification numbers (PMIDs). This term is a parent to *Brain Injuries* in the MeSH hierarchy and also includes terms such as *Spinal Cord Injuries* and *Cerebrovascular Trauma*. The source publications were limited only in requiring that they included neural injury as a topic, with no limitations on journal, species, location, or type of injury. Although this included non-TBI injury and models, (e.g., stroke, spinal cord injury, hypoglossal-nerve injury, etc.), the goal was to undertake as wide a search as possible in order to retrieve remote and ignored possibilities, with the assumption that a significant level of commonality exists between the various forms of injury included under this broad heading in light of their inclusion of common injury pathways such as inflammation and oxidative damage. 99,437 unique citations were returned by this search.

### Semantic predication selection

Semantic predications were extracted from SemMedDB using the PMIDs resulting from the above PubMed search, which yielded 26,441 unique predications. Overall, this set contains 6246 unique concepts, including less informative terms, such as rattus, injury, and patients as well as more specific terms, such as glutamate, brain-derived neurotrophic factor, and methylprednisolone. We then required the predications to contain at least one concept (subject or object) having a UMLS semantic type with potential as a substance biomarker (amino acid sequence; amino acid, peptide, or protein; biologically active substance; body substance; carbohydrate; carbohydrate sequence; chemical; chemical viewed functionally; chemical viewed structurally; eicosanoid; enzyme; gene or gene product; gene or genome; hormone; immunologic factor; inorganic chemical; lipid; neuroreactive substance or biogenic amine; nucleic acid, nucleoside, or nucleotide; nucleotide sequence; organic chemical; organophosphorus compound; receptor; steroid; substance). If only one of the arguments was of this type, the other concept could be of any semantic type. This resulted in the inclusion of some concepts that indicate that the research was performed in animal models such as *Rattus* and *Animals.* We did not discard these nodes because they allow the inclusion of potential biomarkers from basic research in the spirit of translational medicine. Although a given semantic type, for instance “Pharmaceutical Substance”, was not included in the list of target semantic types, it could still appear in a resulting predication if the complimentary subject or object met the requirements. As an example, in the predication *Dexamethasone INTERACTS_WITH NF-kappa B*, the subject, *Dexamethasone*, is of type *Pharmaceutical Substance* and the object, *NF-kappa B*, is of type *Amino Acid, Peptide, or Protein*. This predication qualifies for inclusion because of the object, not the subject. In the predication *Dexamethasone TREATS Rheumatoid Arthritis*, the object, *Rheumatoid Arthritis*, is of type *Disease or Syndrome*, so the predication would not be selected because neither subject nor object is of an included semantic type. After applying this limitation, 18,085 unique predications remained.

### Network of predications

These 18,085 predications extracted from neurological injury MEDLINE citations and containing a potential biomarker as subject or object were then linked together as a network. This network represents all of the known substance activity involved in neurotrauma, as indicated by the semantic predications included in SemMedDB. The nodes of the network represent arguments (subject or object) from the predications, and the edges represent the predicates or relationships between subjects and objects. Each subject-object pair might have multiple predicates. For example, both *Melatonin INHIBITS Free Radicals* and *Melatonin COEXISTS_WITH Free Radicals* may have been asserted in the literature. When counting edges in the network, each predicate between the same subject and object in such predications was counted separately. Additionally, each subject-predicate-object triplet could have been asserted once in MEDLINE (and thus in SemMedDB) or as many as dozens of times. When taking into account each predication extracted from multiple citations, the network has 6246 total nodes and 18,085 total edges. When only unique (different) predications are considered (regardless of the number citations they were extracted from), the number of nodes in the network remains 6246, but the number of edges is 14,085. This is still a rather large network; to reduce it to more manageable size, further filtering was carried out.

### Network filtering: degree centrality

**Figure 3:**
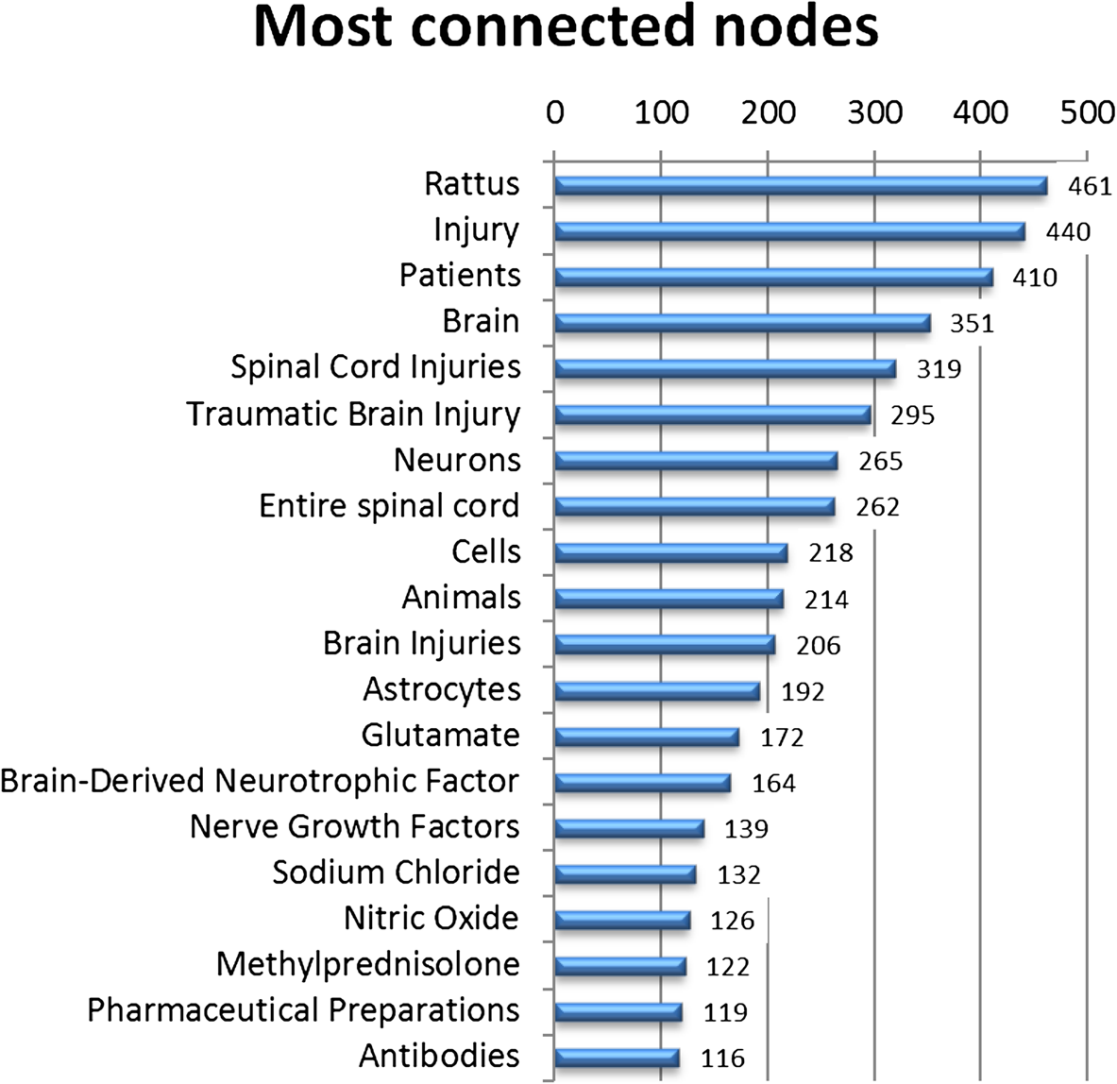
20 most connected nodes in unfiltered network.

**Figure 4:**
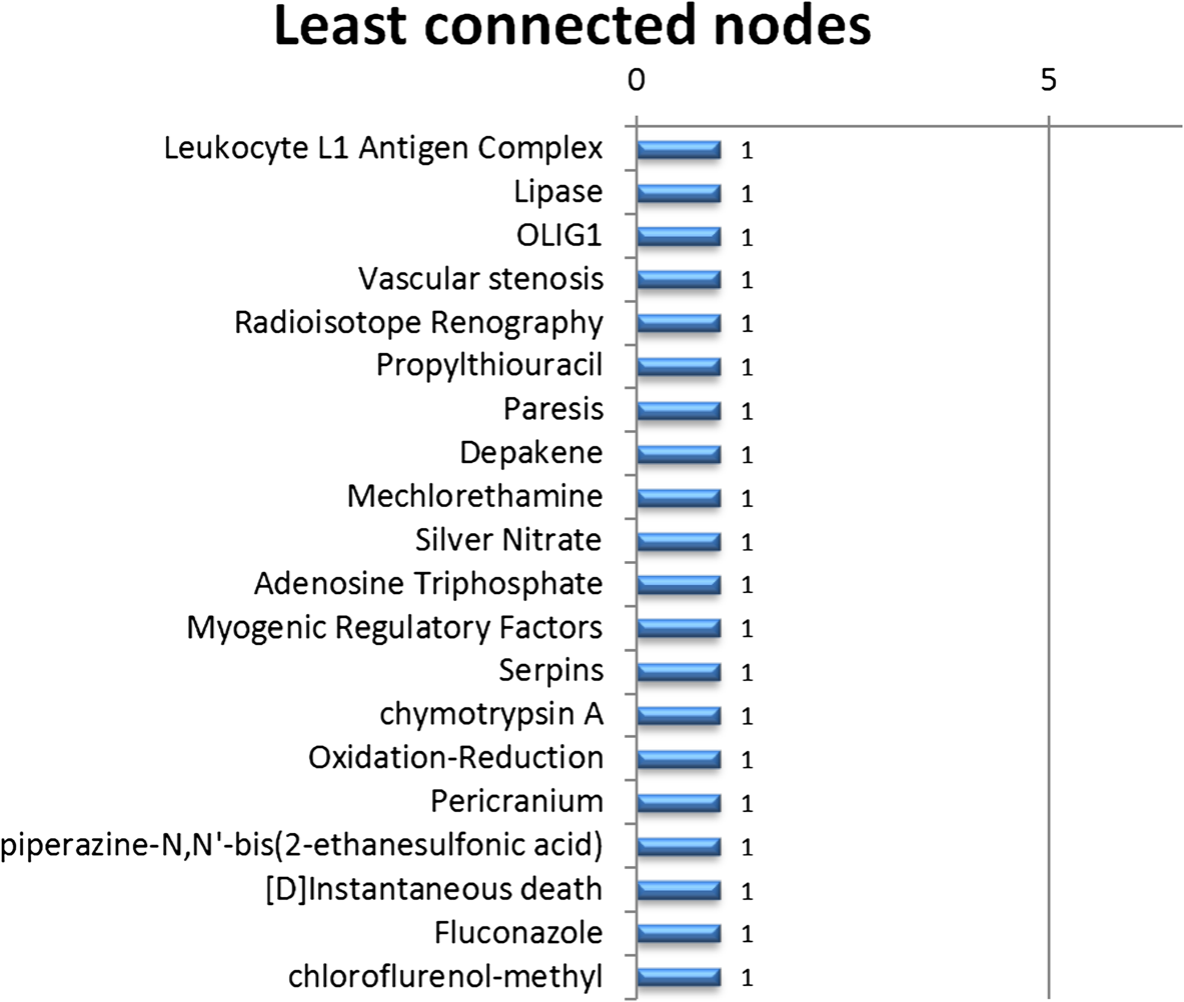
20 from the 2688 nodes with only a single predication in the unfiltered network.

The first cutoff applied was degree centrality. After attempting several thresholds, a node degree cutoff of 0.0000800641 was empirically chosen to provide a network with more than 50 and fewer than 100 nodes, thereby providing a humanly readable graph. This degree correlates to a node having edges connecting to 50 other nodes. For example, the concept *Traumatic Brain Injury* is connected to 295 other nodes with a degree of 0.0004724 and, therefore, is maintained in the network after degree filtering. However, the concept cyclooxygenase 2 is connected to only 43 concepts with a degree of 0.00006886 and so is eliminated. The 20 most highly connected concepts are shown in Figure 3, and 20 examples of the 2688 nodes which had only a single connection are provided in Figure 4.

### Network filtering: frequency of occurrence

**Figure 5:**
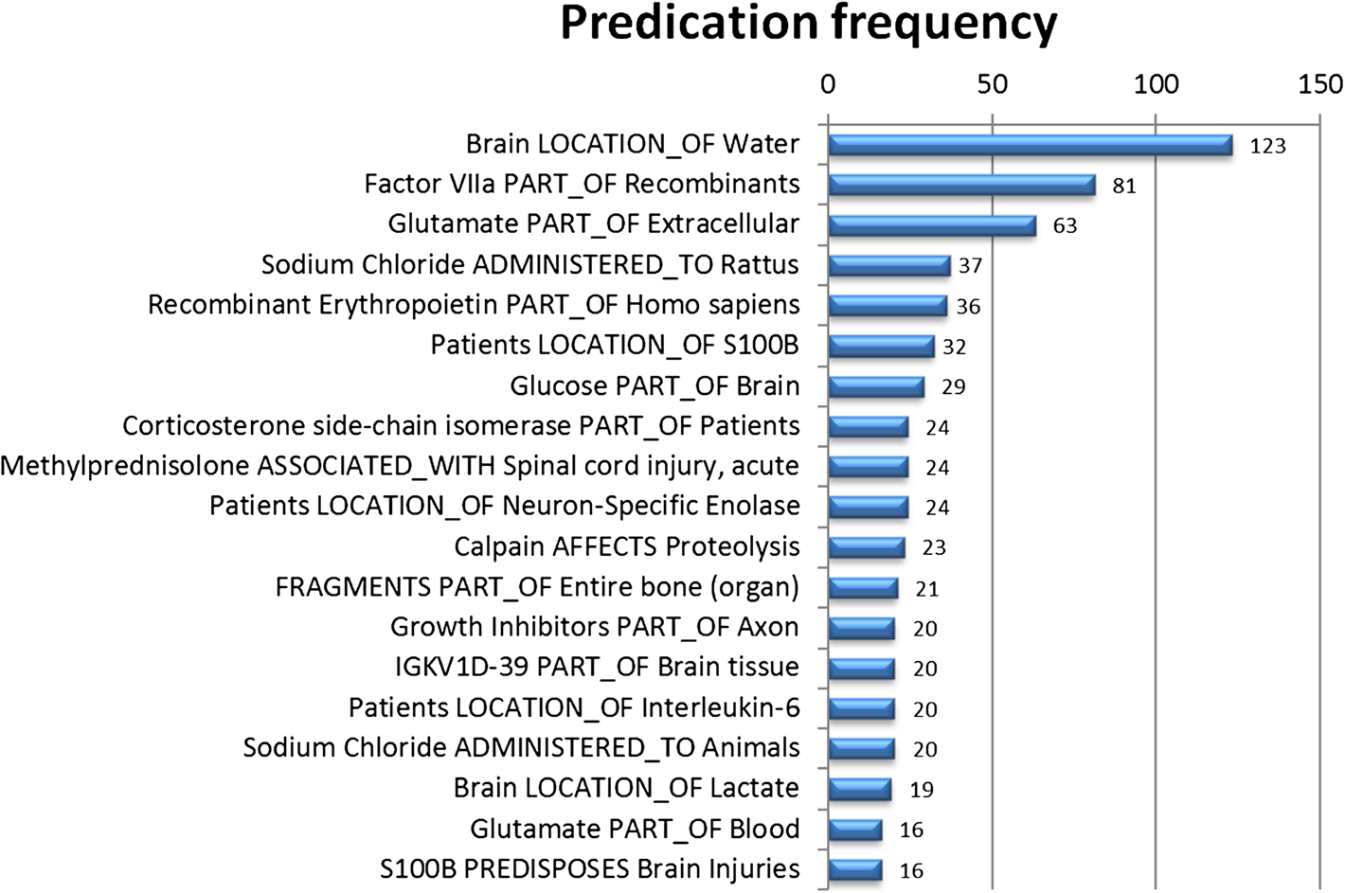
20 most frequent predications in the unfiltered network.

**Figure 6:**
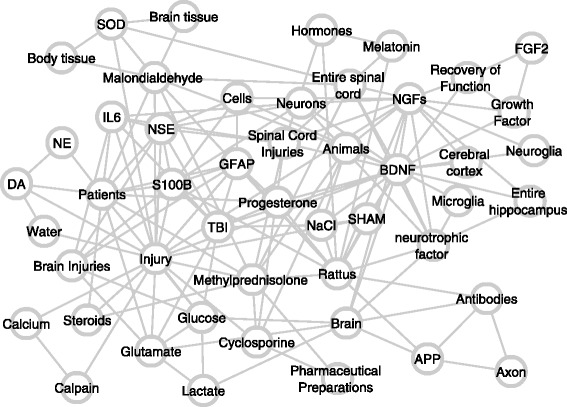
Visualization of substance predication network. The network contains 49 nodes and 1021 edges. Multiple edges between a pair of nodes are represented as a single edge for visual simplicity; therefore edge labels are not included. Abbreviations: FGF2 = fibroblast growth factor 2, NGFs = nerve growth factors, BDNF = brain-derived neurotrophic factor, NaCl = sodium chloride, APP = amyloid-beta precursor protein, SOD = superoxide dismutase, NSE = neuron specific enolase, GFAP = glial fibrillary acidic protein, TBI = traumatic brain injury, IL6 = interleukin 6, NE = norepinephrin, DA = dopamine, SHAM = salicylhydroxamic acid.

Frequency of occurrence was used in conjunction with degree centrality to increase the saliency of the network. A given edge (predicate) between highly connected nodes (arguments) was required to have a frequency of occurrence of 2 in an attempt to eliminate spurious extractions while still including rare statements. As an example, the predication *Interleukin-3 DISRUPTS Cell Death* is maintained in the final network because it occurs twice in the SemMedDB predication set. Because *NADPH Dehydrogenase INTERACTS_WITH Glial Fibrillary Acidic Protein* occurs only a single time, it is not included in the final network. The most frequently occurring predications from this set are provided in Figure 5. This refinement requiring a frequency greater than or equal to 2 and a node degree greater than or equal to 0.0000800641 (50 or more connections) resulted in 1021 predications with 49 unique concepts (see Figure 6).

### Substance network visualization

**Figure 7:**
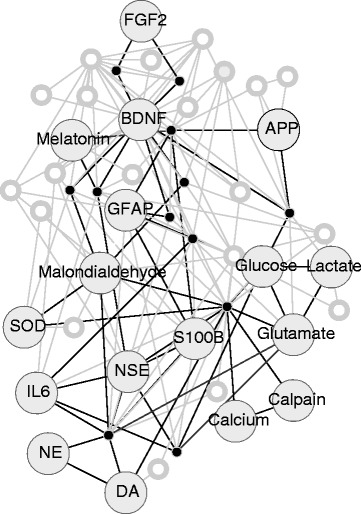
Visualization of interaction network of TBI substances. Only substance nodes are labeled and paths between substance nodes are colored black for lengths one or two edges. Abbreviations: FGF2 = fibroblast growth factor 2, BDNF = brain-derived neurotrophic factor, APP = amyloid-beta precursor protein, SOD = superoxide dismutase, NSE = neuron specific enolase, GFAP = glial fibrillary acidic protein, IL6 = interleukin 6, NE = norepinephrin, DA = dopamine.

The resulting network was visualized as a network in Cytoscape [66]. Redundant edges between connected nodes were reduced to a single edge for visual simplicity. In addition to the substance concepts targeted, it also contains non-substance concepts which are coupled to the substances in the final predication set. An additional network visualization was produced (Figure 7), reformatted to focus on the resulting potential biomarkers. All non-candidate concepts were reduced in size and labels removed. A candidate subnetwork was formed consisting of substance nodes, edges connecting them, and directly intermediate nodes and edges (single nodes between two substances if no edge directly connected the pair). Nodes and edges outside of the candidate subnetwork were also colored gray to further reduce visual prominence.

### Substance network semantic distribution

The final network was analyzed to outline the distribution of UMLS semantic types and predicates. The semantic types of nodes were sorted and tallied as were the predicate for each token of the edges.

### Substance identification precision

SemMedDB maintains a reference to the specific sentence in the original citation that was the source for each predication. Each substance in Figure 6 was compared against this source sentence and evaluated for consistency with the sentence, not for truth value. In other words, we evaluated only whether the substance occurs in the text; whether or not the text provided a biomedically accurate statement was not evaluated at this stage (however, truth value was addressed in Section Evaluation of biomarker potential). Precision was calculated for the resulting substance list using the number of correct substances in the final network and the total number of substances in the final network as follows:

$$ \mathrm{Precision} = \left(\mathrm{Correct}\ \mathrm{Substances}\right)/\left(\mathrm{Total}\ \mathrm{Identified}\ \mathrm{Substances}\right)\ . $$

### Evaluation of biomarker potential

Each of the substances in the final, filtered network was individually reviewed manually as a potential mTBI biomarker. The evaluation was based on 3 questions: is there evidence of a change in the level of this substance during traumatic brain injury, is this change evidenced in blood, and has the substance been previously investigated as a biomarker for traumatic brain injury. We searched PubMed with the query “[*substance name*] AND traumatic brain injury AND (serum OR blood)” and the resulting articles were explored to provide answers to the evaluation questions.

## Results

### Filtered network

There are a total of 17 substances out of the 49 concepts in the final network. The first version (Figure 6) shows all concepts (49) and their connections (145), while in the second (Figure 7), a candidate subnetwork is emphasized in black containing 17 substances as labeled nodes and the 48 edges connecting them. The candidate subnetwork also contains 12 unlabeled non-substance nodes. One node shown in the complete network was incorrectly identified as the substance SHAM (salicylhydroxamate) instead of “sham” (meaning a false experimental action) while the 17 other substances were correctly extracted, for a precision of 0.94.

### Substance network semantic distribution

As seen in Table 1, the most common predicate in the final network is *LOCATION_OF* with 26 instances. This represents 22% of the 209 total edges. The predicate *PREDISPOSES*, which is a clear indicator of biomarker potential, is significantly lower at 12 edges (5.7%). The semantic type *Amino Acid, Peptide, or Protein* was by far most common with 13 out of the 49 nodes (26.5%) as seen in Table 2. This semantic type was also dominant within the subset of substance nodes (Table 3) with 8 of the 17 (47.1%).

**Table 1:** **Predication frequency in final network**

LOCATION_OF	46
ASSOCIATED_WITH	28
PART_OF	25
AUGMENTS	20
PREDISPOSES	12
ADMINISTERED_TO	11
ISA	11
AFFECTS	10
CAUSES	10
COMPARED_WITH	6
DISRUPTS	5
INTERACTS_WITH	5
TREATS	5
STIMULATES	4
INHIBITS	3
COEXISTS_WITH	2
NEG_ADMINISTERED_TO	2
NEG_INTERACTS_WITH	2
PRODUCES	2

**Table 2:** **Semantic type frequency in final network**

Amino acid, peptide, or protein	13
Body part, organ, or organ component	5
Biologically active substance	4
Cell	4
Hormone	4
Injury or poisoning	4
Organic chemical	3
Neuroreactive substance or biogenic amine	2
Animal	1
Cell component	1
Gene or genome	1
Inorganic chemical	1
Mammal	1
Pharmacologic substance	1
Patient or disabled group	1
Sign or symptom	1
Steroid	1
Tissue	1

**Table 3:** **Semantic type frequency of substances in final network**

Amino acid, peptide, or protein	8
Organic chemical	3
Biologically active substance	2
Neuroreactive substance or biogenic amine	2
Gene or genome	1
Hormone	1

### Evaluation of biomarker potential

The results of the substance verification in Table 4 provide an estimate of level of interest for further research as a member in a biomarker panel. In general, the substances show evidence of change in TBI in the literature, with two exceptions: amyloid beta-protein precursor and calpain. (Although calpain itself does not appear in the literature, the calpain-derived NH_2_-terminal fragment of α-spectrin fragment does [67]). Timing and degree of change may also be an issue regarding the effectiveness of some substances as mTBI biomarkers. Reduced levels of calcium appear to return to normal within as little as 4 hours of trauma [68]. Glutamate levels increase in cerebral spinal fluid but there is no evidence for measurable changes in blood [69-72]. And a conflict exists in the literature for melatonin. One study reports a decrease in serum melatonin after TBI [73] while another reports no change in blood but an increase in cerebral spinal fluid [74].

**Table 4:** **Verification of substances in TBI physiology and TBI biomarker research**

		**Changes in trauma?**	**Changes in blood?**	**Previously studied?**	**PMIDs**
1	Brain-derived neurotrophic factor	Yes	Yes	Yes	11585248, 22528282, 20679891
2	Fibroblast growth factor 2	Yes	Yes	Yes	11320217, 7696886
3	Glial fibrillary acidic protein	Yes	Yes	Yes	16266720, 22528282, 21079180
4	Neuron-specific enolase	Yes	Yes	Yes	16716992, 22528282
5	S100B	Yes	Yes	Yes	19257803, 22528282, 21079180
6	Amyloid beta-protein precursor	Yes	No	Yes	8140894, 15258792
7	Interleukin-6	Yes	Yes	No	20850781, 20858121
8	Malondialdehyde	Yes	Yes	No	11280646, 11466564
9	Superoxide dismutase	Yes	Yes	No	17869973
10	Glucose	Yes	Yes	No	20889287, 9808254
11	Lactate	Yes	Yes	No	18183032, 20889287
12	Dopamine	Yes	Yes	No	7584744
13	Norepinephrine	Yes	Yes	No	6886758, 3592639
14	Calcium	Yes	Yes	No	10386980, 4637556
15	Melatonin	Yes	Yes*	No	18183032, 17060154
16	Glutamate	Yes	Yes*	No	20225002, 21878868
17	Calpain	No^+^	No^+^	No^+^	19811094

*Modest change or conflicting reports. ^+^Although Calpain itself does not change in trauma, its products do change and are found in the blood and have been studied.

## Discussion

Most substances identified in this study as worthy of consideration as mTBI biomarkers fall into four general categories: previously studied biomarkers (amyloid beta-protein precursor, brain-derived neurotrophic factor, fibroblast growth factor 2, glial fibrillary acidic protein, neuron-specific enolase, S100b); neurotransmitters (glutamate, dopamine, norepinephrine); inflammation and cell injury markers (interleukin-6, calpain breakdown products, malondialdehyde, superoxide dismutase); and ubiquitous substances (glucose, lactate, calcium).

Although all of the resulting substances were reviewed in depth during the methodology, the following illustrate the information contained in the resulting mTBI biomarker network and the information retrieved during the validation process. These examples suggest possible implications for clinical practice retrieved directly from the research literature.

### Glutamate

The well-known association between glutamate and TBI is present in the network as *Glutamate ASSOCIATED_WITH Traumatic Brain Injury* (PMID 17014847), but relationships that focus on interconnectedness with other substances in the network are also present. For instance, *Lactate INTERACTS_WITH Glutamate* is extracted from (3) which notes that glutamate is produced from the metabolism of lactate in TBI, and perhaps a less familiar relationship, *Glutamate STIMULATES Lactate* is extracted from (4), highlighting glutamate’s role in activating lactate production in a potentially neuroprotective, estrogen receptor-dependent manner.

(3) Infusion with … 3-(13)C-lactate produced (13)C signals for glutamine … indicating tricarboxylic acid cycle operation followed by conversion of glutamate to glutamine. (PMID 19700417)
(4) These results suggest a new neuroprotective mechanism of 17beta-estradiol by activating glutamate-stimulated lactate production, which is estrogen receptor-dependent. (PMID 11368971)

### Glucose and lactate

Glucose and lactate are substances within the network that (along with calcium) are ubiquitous in the human system. Within the context of TBI a major concern is the decrease of available glucose in the brain due to ischemia and the subsequent increase in lactate. This is included in our neural injury network as *Glucose COEXISTS_WITH Lactate*, which is extracted from (5). Sentence (6) is another source of the link between lactate and glucose, but the source sentence provides the additional knowledge that peripheral blood glucose levels are not isolated from cerebral levels and lactate production in the TBI brain, while (7) presents the opposite, that arterial lactate is connected to cerebral lactate and subsequently cerebral glucose, represented in our network as *Lactate COEXISTS_WITH Glucose*. As suggested in (4) above, the ratio of glucose to lactate is influenced by glutamate. It has been suggested that this may be a result of astrocytes responding to increased extracellular glutamate by increasing glycolysis and, thereby, lactate production [75].

(5) Following TBI, neuron use initially increases, with subsequent depletion of extracellular glucose, resulting in increased levels of extracellular lactate and pyruvate. (PMID 18826359)
(6) Arterial blood glucose significantly influenced signs of cerebral metabolism reflected by increased cerebral glucose uptake [and] decreased cerebral lactate production… (PMID 19196488)
(7) We conclude that arterial lactate augmentation can increase brain dialysate lactate, and result in more rapid recovery of dialysate glucose after FPI [fluid percussion brain injury]. (PMID 10709871)

### Biomarker panels

Although there have been a limited number of attempts to include multiple biomarkers in panels for TBI [67,76,77], these have not included some of the types of substances returned in our results. To a large degree the absence of consideration for such substances may be explained by their lack of specificity or their ubiquitous nature. The level of specificity as an analyte for these neglected substances is significantly higher for an individual marker to stand on its own, and substances that are frequent if not ubiquitous in normal physiology are not obvious as candidates for TBI identification. Taken on their own, glucose and calcium levels are not useful as measures of brain injury. However, a panel of markers could better represent the complex network of molecular changes that occur during TBI and change the goal from an individual marker/single variable to a panel ameliorates the lack of specificity – as long as the panel as a whole provides adequate sensitivity and specificity.

### Limitations of study

These resulting data provide a clinically relevant hypothesis of potential mTBI biomarkers, which requires experimental validation. In our investigation into the validity of the results, it was evident that for some of the substances, especially the previously-studied biomarkers, the background TBI model-based studies have already been completed. For others, this is not the case and basic exploration in models may need to be pursued before moving towards clinical research.

The current result set is limited to the uppermost extreme of node connectedness and therefore potentially overlooks less investigated substances that appear in fewer publications. An elimination of the most frequent predications may enrich the result set for substances less familiar and thereby, potentially, more valuable. The current threshold is principally set to provide a visually comprehensible network in the result, though such a visualization is not required. Reducing the threshold for inclusion would expand the list with significant compounds, including microRNA.

**Figure 8:**
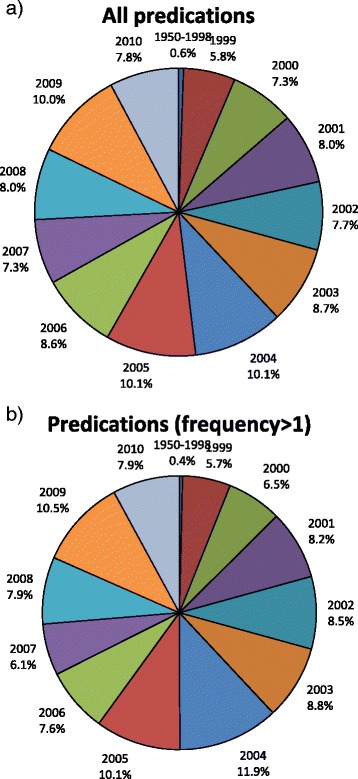
The relative distribution of predication frequency by year. **a)** All frequencies. **b)** Predications that have at least 2 occurrences. 78.8% of predications occurred only once.

When we filter by frequency of occurrence with a cutoff of 2 instances we eliminate 78.8% of the predications. This step risks eliminating predications that occur only once because they are completely new and have only been stated once. Figure 8a shows that 7.8% of predications were from citations in 2010. As seen in Figure 8b, when all predications that occur only one time are removed, the 2010 fraction increases to 7.9%. This shows that there is not a disproportionate elimination of predications from the most recent citations and the loss of unique predications due to their novelty likely plays a much less significant role than the elimination of inaccurate extraction by SemRep. On the other hand, as SemRep precision continues to improve, additional attention to date of publication may be required.

### Future directions

Creating a map of neural injury interactions offers significant potential for basic science research. Additionally, our refinement of the network to identify the most significant interactions according to their degree centrality and frequency facilitates the quick translation of published research data into clinical practice. The resulting compound list is clearly interesting in the context of clinical applicability and merits further study. This technique allows the investigation of potential biomarkers to be focused, potentially reducing the wet-lab effort and reducing the time of assay development.

Now that we have outlined a basic methodology, we would like to compare this method with various other methods combining information extraction and network analysis to understand the advantages and disadvantages to different approaches.

Our current methodology can be expanded as noted above to include different subsets of substances in the final result. Additionally, this methodology is not limited to biomarker discovery but can also be applied to other areas of medical discovery, including novel therapeutic targets, drug repurposing, and others.

## Conclusion

We have explored the creation of a molecular interaction network that represents neural injury and is composed of semantic predications automatically extracted from the literature. We achieved our goal of providing substances with potential as biomarkers to support the diagnosis of mTBI. The methodology is based on a network of semantic predications representing the interaction of substances observed subsequent to neural insult. Combining semantic predications of TBI substance interactions into a network in this way correlates well with systems biology (and by extension, systems medicine), which is concerned with the complex network interplay of a biological unit and represents injury and illness as a perturbation to the network.

Predications were extracted by SemRep and the component subject or object concepts were mapped to nodes and their relationships (predicates) mapped to edges, creating a network of relations. This network represents a summary of the physiological and pharmacogenomic space of neurological injury, as presented in the literature included in MEDLINE. To identify clinically significant candidates for mTBI biomarkers, the network was then filtered by degree centrality and frequency, greatly reducing the density of concepts and relationships. The resulting network produced 17 compounds to be considered as mTBI biomarkers, both previously investigated and novel as TBI biomarker candidates. The interaction of several of these is discussed as the basis for a panel of biomarkers to more effectively diagnose mTBI than is currently possible.

### Availability of data and software

The predication data (SemMedDB) is available at skr3.nlm.nih.gov. Degree and frequency filtering java programs are available at skr3.nlm.nih.gov/mTBI.
